# Personalized checkpoint acupuncture can reduce postoperative pain after abdominal surgery—a STRICTA-conform pilot study

**DOI:** 10.1007/s00423-023-03051-8

**Published:** 2023-10-10

**Authors:** Erfan Ghanad, Cui Yang, Christel Weiß, Mario Goncalves, Maria Joao Santos, Nuno Correia, Christoph Reissfelder, Henry Johannes Greten, Florian Herrle

**Affiliations:** 1 Department of Surgery, University Medicine Mannheim, Medical Faculty Mannheim, University of Heidelberg, Theodor-Kutzer-Ufer 1-3, 68167 Mannheim, Germany; 2 Department of Medical Statistics and Biomathematics, Medical Faculty Mannheim, Heidelberg University, Mannheim, Germany; 3 Heidelberg School of Chinese Medicine, Heidelberg, Germany; 4 TCM Research Centre, Piaget Institute, Gaia, Portugal; 5 Department of Surgery, Prien Hospital on Chiemsee, Prien am Chiemsee, Germany

**Keywords:** Postoperative pain, Acupuncture, ERAS®, Analgesia, Chinese medicine

## Abstract

**Background:**

Optimal pain management is one of the core elements of Enhanced Recovery After Surgery (ERAS®) protocols and remains a challenge. Acupuncture (AC) is an effective treatment for various pain conditions. Systematic and personalized allocation of acupoints may be decisive for efficacy.

**Methods:**

Based on the predominant pressure sensitivity of six gastrointestinal (GI) checkpoints (G1-G6), we devised a method to detect personalized patterns of pain and a corresponding set of acupoints. We performed a single AC treatment with semi-permanent needles and assessed the visual analogue scale (VAS) score, pain threshold based on pressure algometry (PA), and temperature changes on abdominal skin areas before and 5 min after AC.

**Results:**

Between April and June 2021, thirty-eight patients were prospectively included in this pilot study. The mean reduction in subjective pain sensation as assessed by VAS was 86%, paralleled by an augmentation of the pain threshold as measured by PA by 64%. A small but significant increase in the skin temperature was observed above the abdominal surface. These effects were independent of the type of surgery.

**Conclusion:**

Checkpoint acupuncture may be a complementary tool for postoperative pain management. Further investigations are needed to explore this analgesic effect.

**Supplementary Information:**

The online version contains supplementary material available at 10.1007/s00423-023-03051-8.

## Introduction

Postoperative pain is one of the major complaints [1, 2] and fear in patients undergoing surgical interventions [3]. Optimal perioperative pain management within optimized perioperative pathways, such as enhanced recovery after surgery (ERAS®), remains challenging. Postoperative pain frequently impedes compliance with ERAS®-core elements like early mobilization and gastrointestinal (GI)-recovery [4, 5]. According to a US national survey, the majority of patients receiving analgesic medications to reduce postoperative pain reported adverse effects, such as vomiting and nausea. In addition, this study also demonstrated that 39% of the patients showed no adequate response to their first dose of analgesic treatment and that they complained of consistent moderate to severe pain after their initial dose [6]. In a cross-sectional study, almost 90% of perioperative patients experienced moderate-to-severe fear of postoperative pain [3]. Recent studies showed that perioperative fears have a negative impact on the surgical outcome as well as the postoperative recovery [7, 8]. Furthermore, poor postoperative pain management could facilitate the development of chronic pain and opioid dependence [9], leading to increased morbidity and impaired quality of life [10, 11].

Acupuncture (AC) has become an increasingly popular modality for the treatment of acute and chronic pain [12]. Additionally, acupuncture significantly improved gastrointestinal function and reduced postoperative hospitalization [13]. A contemporary concept of AC [14–17] understands it as a vegetative reflex therapy. According to this explanatory model, ancient diagnostic systems are traditionally applied to determine the vegetative functional state and to choose an individually effective set of AC-points. This diagnostic approach, consisting of observation, auscultation, olfaction, and palpation, is highly experience-based [18], time consuming, and difficult for non-acupuncturists. Hence, we investigated a method to address abdominal discomfort and pain through palpation of six specific abdominal points: gastro 1–gastro 6 (G1-G6) without making use of the ancient diagnostic approach.

## Materials and methods

### Study design

The study was designed as a prospective proof-of-principle study investigating the analgesic effects of checkpoint AC in patients after abdominal surgery. Informed consent was obtained before enrollment, according to a clinical trial protocol approved by the local Ethical Committee (EK 2021-604).

### Eligibility criteria

Adult patients who underwent elective or emergency abdominal surgery with a postoperative pain score of ≥3 on a 10-point visual analogue scale (VAS) were eligible. Sufficient language communication skills were necessary for inclusion in the study. Patients with needle phobia, relevant actively treated psychiatric conditions such as bipolar disorder, chronic pain syndrome prior to surgery, polyneuropathy, relevant bleeding disorders, impaired mental state, and poor German language communication skills were excluded.

### Standard patient care

All participants underwent standard pain management according to the ERAS® protocol. On surgical ward, patients were given scheduled baseline analgesics, such as paracetamol or dipyrone (1g every 6 h). Oxycodone with naloxone (oral 20 mg/10 mg every 12 h) was used as the first-line rescue medication. If no adequate pain relief was achieved, immediate release oxycodone was prescribed (10 mg upon request).

Patients who underwent bowel followed a highly standardized Bowel-ERAS®-Protocol within our ERAS®-qualified department. Other patients followed local, highly standardized clinical pathways integrating the ERAS®-variables [19].

### STRICTA criteria

Study reporting was conducted according to the STRICTA guidelines [20]:

1. Acupuncture rationale: Acupuncture was performed based on our established checkpoint concept (G-points) [21, 22], which dates back to reflections described in the Shang Han Lun by Zhang Zhongjing before 220 AD [23].
2. Needling technique: length, diameter, pressure of insertion, and depth of insertion were identical by using semi-permanent needles (Sedatelec ASP Original Classic steel needle) [24, 25]. We did not seek for subjective needling sensations (de qi) or any other individual responses.
3. All patients received a single AC treatment after surgery. The needles remained until discharge. Verbal communication was reduced to a minimum.
4. Other treatment components: No additional treatment was administered.
5. The study acupuncturists (EG, JG) performed acupuncture on a daily basis for several years.
6. Control or comparator interventions: As this was a preliminary pilot study focusing on feasibility and practicality, no control group was included.

### Checkpoint acupuncture

**Fig. 1:**
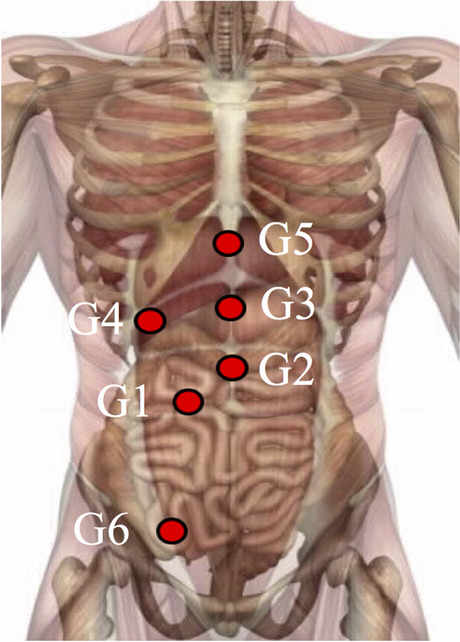
Locations of visceral checkpoints G1–G6: G1 is located above the sphincter Oddi. G2 is above the pylorus (G2). The gastric fundus (G3) and cardia (G5) are located at the midline. G4 is based on the subcostal space on the midclavicular line (corresponding to the gallbladder). G6 marks the transition from the small to the large intestine

**Fig. 2:**
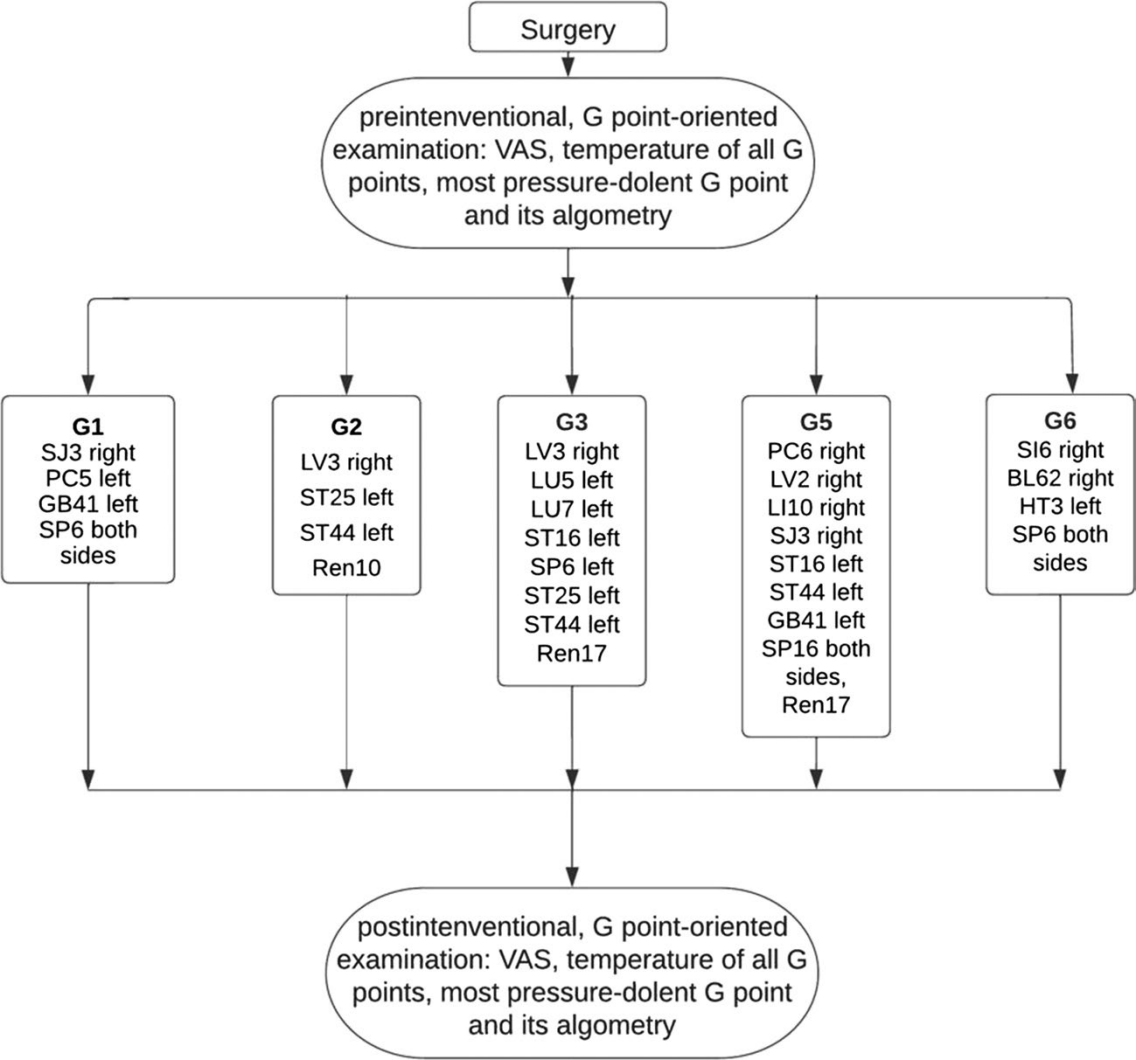
Flow chart of the study, including standardized sets of acupoints for each syndrome. HT: heart; LU: lung; PC: pericardium; SI: small intestine; LI: large intestine; SJ: triple burner; LV: liver; KI: kidney; SP: spleen; ST: stomach; BL: urinary bladder; GB: gallbladder; Ren: Conception Vessel meridian

AC was applied as an additional element in the standardized multimodal approach to relieve pain after abdominal surgery. In this study, we examined six defined regions located in the abdominal cavity (Fig. 1, Tables 1 and 2) [21, 22]. Each point was palpated, and the most sensitive abdominal pressure point was manually identified (similar to examining the McBurney point for appendicitis). This hypersensitive region may resemble a dysfunctional vegetative pattern that can be addressed using defined AC strategies (Fig. 2).

**Table 1:** Location of G1–G6 points and their according anatomic structures

G-point	Location—abdominal wall	Region of interest (abdominal cavity)
G1	2 cun left to the umbilicus	Sphincter Oddi
G2	2 cun above the umbilicus	Pylorus
G3	Halfway between the xiphoid process and the umbilicus	Gastric corpus
G4	On the gallbladder, comparable to Murphy sign	Gallbladder
G5	Epigastric angle	Antrum and his angle
G6	Above the IC-valve	Terminal ileum

*cun* The width of a patient’s thumb at the knuckle; *IC* ileocecal

**Table 2:** Baseline patient characteristics

Baseline characteristics	Numbers
Sex	
Male	17 (45%)
Female	21 (55%)
Age	50.9±17.1
Surgeries	
Minimal invasive (%)	28 (74%)
Open (%)	10 (16%)
Elective surgery (%)	33 (87%)
Emergency surgery (%)	5 (13%)
Postoperative day of AC:	
On day of surgery (%)	10 (26%)
POD 1 (%)	18 (47%)
POD 2 (%)	9 (24%)
POD 4 (%)	1 (3%)
Days until surgery following admission	Median 1 (0–4)
Days until AC following admission	Median 1 (0–21)
Length of hospital stay (days)	Median 3 (2–37)
Days until discharge following AC	Median 2 (1–16)
Complications after surgery	3 (8%)

### Study assessments and intervention

#### Examination 1

After verifying the inclusion and exclusion criteria, VAS was assessed. The temperature was measured on all six visceral indicator points (G1–G6) using a touchless infrared thermometer (Domotherm Free, NT17, CE-approved). The most pressure-sensitive checkpoint was detected by careful palpation, and the pain threshold was assessed via digital PA (PCE instruments-FM 200 device) [26, 27].

#### Intervention

Patients were treated with AC points selected according to checkpoint diagnosis (Table 2, Fig. 2).

#### Examination 2

Following a 5-min resting period, all parameters of Examination 1 were re-assessed.

### Statistical analysis

SAS software (version 9.4; SAS Institute Inc., Cary, NC, USA) was used for all statistical calculations. For qualitative factors, absolute and relative frequencies are given. Quantitative variables approximately normally distributed are presented by mean value and standard deviation (i.e., temperature). For skewed or ordinal scaled data, median and range are given (i.e., VAS). Graph Pad Prism (Version 9.4.1) was used to create the figures. In order to compare parameters before and after intervention, a Wilcoxon test or a *t* test for two paired samples was used, as appropriate. For the comparison of two independent subgroups, Wilcoxon two-sample test was performed. The results of the statistical test were considered statistically significant at *p*<0.05.

## Results

Between April and June 2021, thirty-eight patients were included in this pilot study. Twenty-one (55%) were female, and 17 (45%) were male. Mean age was 50.9±17.1 (23-80) years. Thirty-three patients (87%) underwent elective surgery, and five patients (15%) underwent emergency surgery (Table 2). The details of the procedures are listed in Supplementary Table 1. Three (8%) patients developed complications after surgery (two anastomotic insufficiencies and one urinary retention).

### Total cohort

#### Subjective pain assessment by VAS

**Fig. 3:**
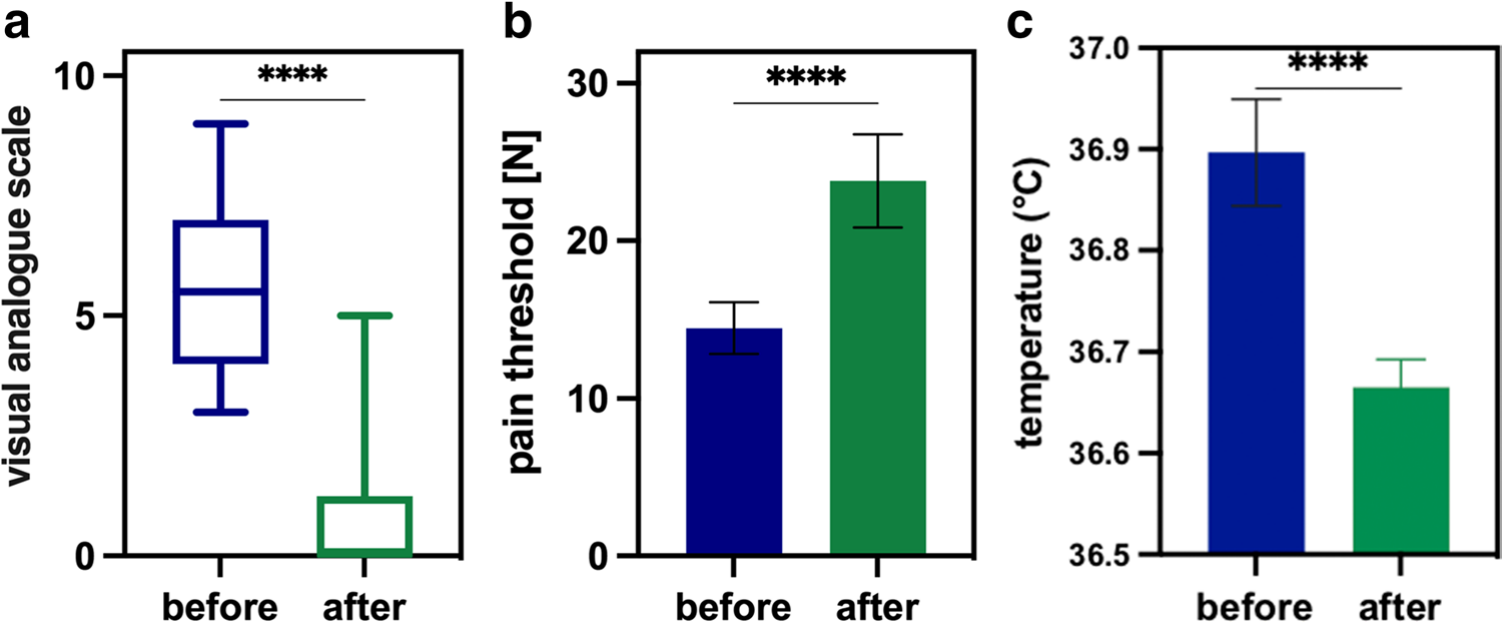
Pain via VAS (**a**), algometry (**b**), and skin temperature (**c**) before (blue) and after acupuncture (green), showing a highly significant difference between these groups (****p<0.0001). Whiskers: minimum to maximum

Before AC, the median VAS score for all 38 patients was 5.5 (3–9) indicating significant pain. Following acupuncture, the median VAS score was 0 (0–5) with an average reduction by 86% (25–100%, (*p*<0.0001). Figure 3A shows the overall effect of acupuncture on the pain score (VAS). Complete pain remission was achieved in more than half of the cases (55.5%) after single AC treatment.

#### Objective pain assessment by PA

In addition to subjective pain relief, AC augmented the pain threshold. The median pain threshold before treatment was reached at a pressure of 12.8N (1.78N–41.3N), compared to 21.8N (2.0N–79.9N) after AC, indicating a clinically relevant pain reduction and augmentation of the pain threshold by 67% on average. Hence, a highly significant difference was detected between the algometric measurement before and after treatment was detected (*p*<0.0001, Fig. 3B).

### Skin temperature

With means of 36.9°C ±0.4°C before and 36.7°C ±0.2°C after the intervention, a significant difference has been measured above G1 (*p*<0.0005, Fig. 3C). Also, for G2 and G6, significant decreases were observed (G2: 36.9°C ±0.3°C before and 36.7°C ±0.2°C after AC, *p* = 0.0009; G6: 37.0°C ±0.4°C before and 36.5°C ±0.5°C after AC, *p* = 0.0099). No differences were observed above other G-points.

### Effect over time

**Fig. 4:**
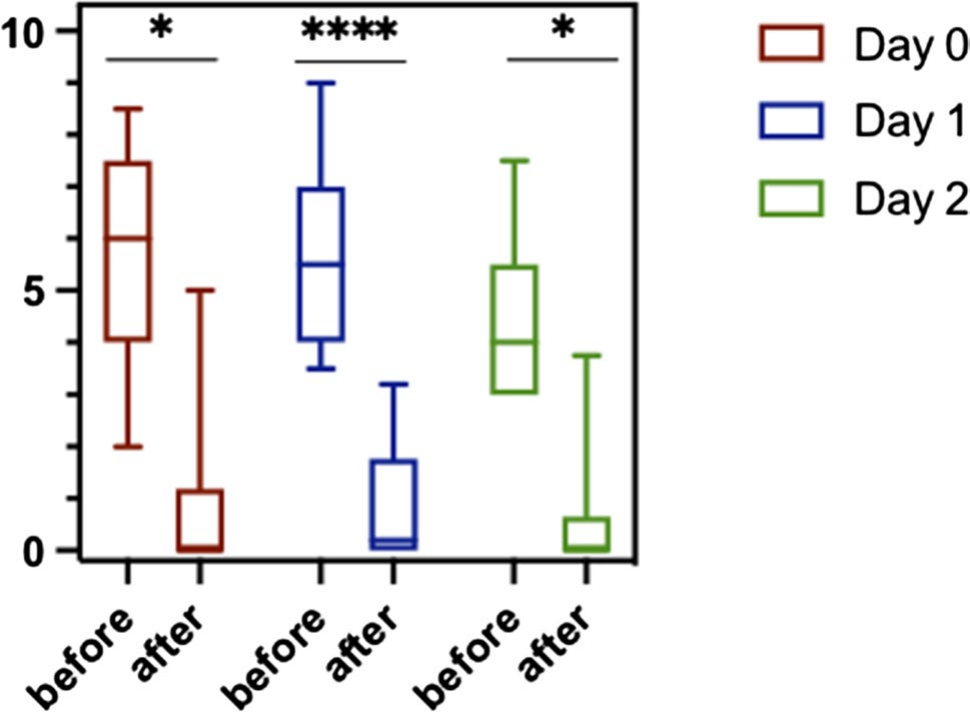
Effect of acupuncture on different postoperative days. The first column represents the pain score before acupuncture and the second column represents pain levels after acupuncture. Ten patients were treated on the day of surgery, 18 on POD 1, and nine on POD 2. Only one patient was treated on POD 4 (data not shown). On operation day: *p = 0.0195, day 1 postoperative: ****p < 0.0001, day 2 postoperative: *p = 0.0313

As shown in Fig. 4, significant pain reduction was achieved through acupuncture regardless of the postoperative day.

### Subgroup analyses

#### Conventional open vs. minimally invasive laparoscopic

**Fig. 5:**
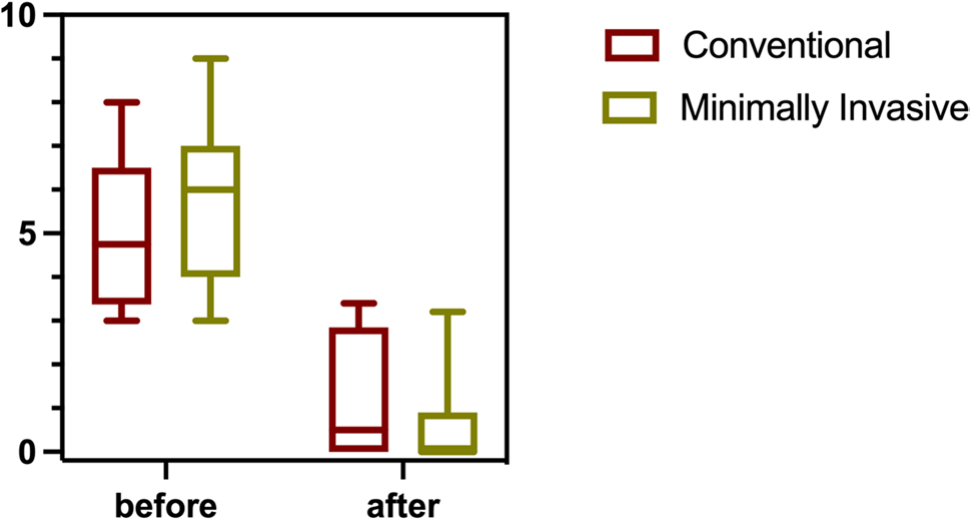
The effect of acupuncture on pain for open surgery (red) and minimally invasive procedures (green) are shown. Acupuncture led to relevant pain release in both groups. No difference in the effect of acupuncture was observed between surgical approaches (*p* = 0.4145). Whiskers: minimum to maximum

AC had similar pain-releasing effects without a significant difference (Fig. 5) between patients undergoing minimally invasive and conventional open surgeries, with median values of 5.75 (3–9) and 4.75 (3–8), respectively (*p* = 0.4145).

#### Distribution of surgery type and G-point-syndrome

Analysis of subgroups revealed that the type of surgery was linked to a tendency towards certain checkpoint syndromes. The largest subgroups included bariatric and colorectal surgeries (supplementary Table 2, 3, 4).

#### Bariatric surgeries (n=16)

##### Subjective pain assessment by VAS

**Fig. 6:**
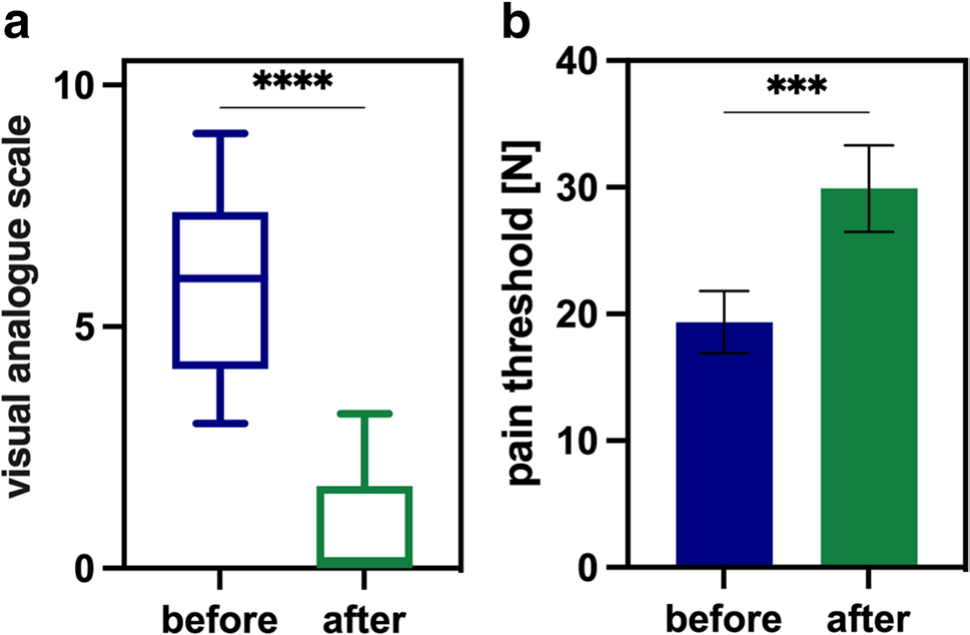
Subtype analysis for bariatric surgeries showing the pain level via VAS (**a**) and algometry (**b**) before (blue) and after acupuncture (green) (***p<0.001; ****p<0.0001)

Overall, patients undergoing bariatric surgery showed reduced pain levels by 86.7% on average, with a median VAS score of 6 (3–9) before and 0 (0–3) after acupuncture (*p*<0.0001), as shown in Fig. 6A. Most of the bariatric patients showed G3-syndrome (compare Supplementary Material Table 2).

##### Objective pain assessment by PA

The initial pain threshold increased from 17.4N (7–41.3) to 29.3 N (10.6–29.7; *p*=0.0003; Fig. 6B). These results demonstrated an equivalent increase in the pain threshold by 54%.

##### Skin temperature

After AC, the bariatric patients showed slight but significant lower temperature levels than before (before: 36,9°C ±0.4°C, after: 36.7°C ±0.2°C; *p*=0.0200 and 36.8°C ±0.2°C and 36.7°C ±0.2° C, *p* = 0.0218) above G1 and G2. No differences were observed above the other G-points.

#### Surgery of the small intestine (n=7)

##### Subjective pain assessment by VAS

**Fig. 7:**
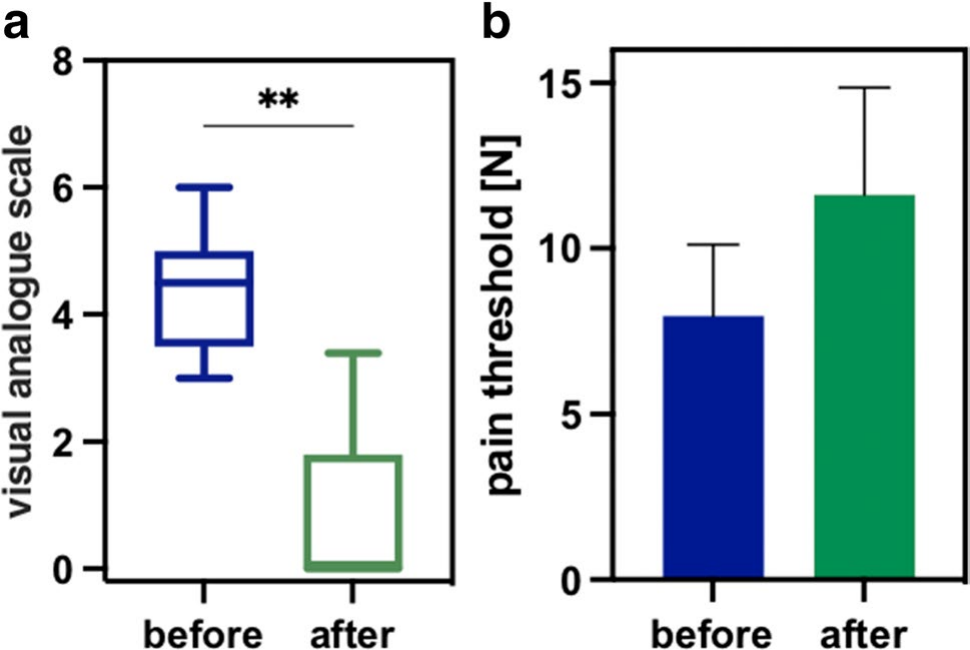
Pain via VAS (**a**) and algometry (**b**) before (blue) and after acupuncture (green) in patients undergoing surgeries of small intestine (**p>0.01)

Median postoperative pain after ileostoma relocation and small intestine resections was 4.5 (min: 3; max: 6), indicating a moderate pain. Through AC, a significant pain reduction of 80% was achieved, reaching a median VAS of 0 (min 0; max: 3,4, Fig. 7A). For checkpoint diagnosis, compare Supplementary Material Table 3.

##### Pain as assessed by algometry

The initial pain threshold (median) increased from 4.1N (2.6N–12.8N) to 6.7N (3.6N–21.6N) after. This results slightly failed to reach statistical significant (*p* = 0.0625, Fig. 7B).

##### Skin temperature

For G1 and G4, slight but significant temperature changes could be observed for this subgroup (each 36.8±0.2°C before and 36.7±0.2°C after AC, *p* = 0.0046 and *p* = 0.0341, respectively).

#### Colorectal surgeries (n=10)

##### Subjective pain assessment by VAS

The median postoperative pain score was 5.5 (min: 3; max: 8). After AC, the patients experienced pain of a median VAS 0 (min: 0; max: 3) equaling a pain reduction of 93% percent on average. Eight patients showed a complete pain remission (VAS 0). Pain reduction was statistically significant (*p*=0.0020). Nine out of ten patients showed either G1- or G3-syndrome (Supplementary Material Table 4).

##### Objective pain assessment by PA

**Fig. 8:**
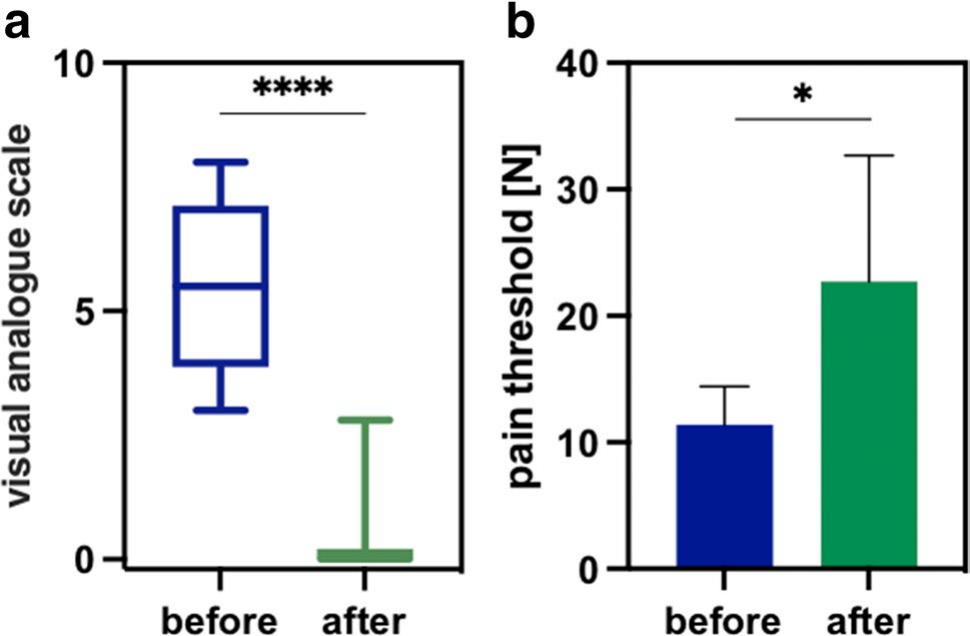
Pain via VAS (**a**) and algometry (**b**) before (blue) and after acupuncture (green) in patients undergoing colorectal surgeries (*p<0.05; ****p<0.0001)

Before AC, the median pain threshold was 10.6N (1.78–25). After AC, an augmented pain threshold of 15.4N (2N–79.9N) could be observed (*p* = 0.0156) (Fig. 8A and B).

##### Skin temperature

Temperature changes above the indicator points were not significantly different (each *p*>0.05).

## Discussion

This present pilot study evaluated the effect of the novel concept of checkpoint AC after abdominal surgery within the ERAS® setting. Significant pain relief after a single acupuncture session was demonstrated in this heterogeneous patient population on different postoperative days and time points. This indicates a potential improvement in postoperative pain management within ERAS® protocols [28].

ERAS® pathways and protocols have emerged over the past 10 years as the gold standard for improving postoperative recovery, resulting in shortened hospitalization and reduced costs [29]. Multimodal analgesia (MMA) is an essential component of ERAS®. Although acupuncture has been proven to be effective in promoting gastrointestinal function recovery and preventing prolonged postoperative ileus [30, 31], its efficacy as complementary analgesic therapy after surgery is controversial [32–35].

Considering that postoperative pain is one of the main concerns of patients undergoing surgery [3], this additional analgesic tool might reduce perioperative fears. Depression and anxiety are psychological elements that appear to have an impact on both the experience of pain and effectiveness of analgesic therapy [7, 8]. Acupuncture has proven to be an efficient treatment for anxiety and depressive disorders [36–40]. This may have amplified the analgesic efficacy observed in our study.

Our data suggest that there may be a correlation between surgical intervention and affected hyperalgesic abdominal pressure points. While most lower GI surgeries had G1-syndrome, bariatric surgeries were prone to have G3-syndrome [22]. G3 is located above the gastric corpus. Hence, a correlation between anatomical location and the respective G-syndrome may be observed.

Pain reduction after checkpoint acupuncture demonstrated a pain-reducing effect that was not influenced by the type of surgery, whether open or minimally invasive. This implies that it may be more effective in alleviating visceral pain than wound pain.

### Limitations

Owing to the pilot design with heterogeneity of patients, surgeries, and intervention time points, the trial was neither randomized nor blinded and prone to selection, performance, and detection bias. Thus, a placebo effect with similar pain reduction effects cannot be eliminated [41]. However, data of the current study can be used for a sample size calculation for future randomized, blinded trials to validate the effect of checkpoint acupuncture after abdominal surgery. Moreover, the endpoints were assessed 5 min after acupuncture; therefore, long-term effects and adverse effects after the observation period remain unclear. They are to be evaluated in further trials.

## Conclusion

This pilot study showed that checkpoint acupuncture may be an effective and safe complementary tool for postoperative pain management, even within the implemented ERAS® pathways. Breaking down the complexity of the diagnosis of Chinese Medicine to a few abdominal checkpoints will allow others to apply AC without requiring generous knowledge of traditional Chinese Medicine. Further randomized, blinded trials are needed to verify these conclusions.

### Supplementary information

ESM 1 (DOCX 73 kb)

## Data Availability

Data can be shared by reasonable request.
