# Supplementary material for: Personalized checkpoint acupuncture can reduce postoperative pain after abdominal surgery—a STRICTA-conform pilot study
Source: Langenbecks Arch Surg. 2023 Oct 10;408(1):391. doi: 10.1007/s00423-023-03051-8 (PMC10562323; doi:10.1007/s00423-023-03051-8)
Supplement: Supplementary file 1 — (DOCX 73 kb) [file 423_2023_3051_MOESM1_ESM.docx]

**Supplementary material:**

**Table 1:** List of included surgery

|  | Surgery performed | Numbers of patients included | Laparoscopic |
| --- | --- | --- | --- |
| Bariatric surgery | Roux-en-Y gastric bypass  Sleeve gastrectomy  Mini bypass | 7  8  1 | 7  8  1 |
| Small intestine | Ileostoma relocation  Small bowel resection | 5  2 | 0  1 |
| Colorectal | Colostoma relocation  Right hemicolectomy and small bowel resection  Transverse resection  Sigmoid resection  Subtotal colon resection  Colectomy  Proctocolectomy  Rectum resection  Rectal rectopexy | 1  1  1  2  1  1  1  1  1 | 1  1  0  1  0  1  1  1  1 |
| Other abdominal surgeries | Esophagus resection  Laparoscopic cholecystectomy | 1  2 | 1  2 |
|  | Stomachal fistula repair after RYGB | 1 | 1 |
|  | Sublay-mesh hernia repair | 1 | 0 |

**Table 2:** Functional assessment by G-point diagnosis for bariatric surgery.

| **Bariatric surgery**  **(n=16; 42%)** | **Type of surgery** | **G-point diagnosis** |
| --- | --- | --- |
|  | RYGB (n=7) | G3: n=7 |
|  | VSG (n=8) | G2: n=2  G3: n=6 |
|  | Mini bypass (n=1) | G5: n=1 |

**Table 3:** Functional assessment by G-point diagnosis for surgeries of the small intestine.

|  | **Type of surgery** | **G-point diagnosis** |
| --- | --- | --- |
| **Surgeries of the small intestine**  **(n=7; 18%)** | Small intestine resections (n=2) | G1: n=1  G6: n=1 |
|  | Stoma relocations (n=5) | G1: n=5 |

**Table 4:** Functional assessment by G-point diagnosis for colorectal surgeries

| **Colorectal surgeries**  **(n=10; 26%)** | **Type of surgery** | **G-point diagnosis** |
| --- | --- | --- |
|  | Colon resection (n=5)  1 patient included with additional small intestinal resection | G1: n=3  G3: n=2 |
|  | Colostoma relocation (n=1) | G1: n=1 |
|  | Sigmoid resections (n=2) | G1: n=2 |
|  | Rectal rectopexy (n=1) | G1: n=1 |
|  | Rectal resection (n=1) | G6: n=1 |

**Table 5:** Functional assessment by G-point diagnosis for different surgeries

| **3**  **Others**  **(n=5; 26%)** | **Type of surgery** | **G-point diagnosis** |
| --- | --- | --- |
|  | Esophagus resection (n=1) | No sensitive G-point |
|  | Cholecystectomy (n=2) | G1: n=1  G2: n=1 |
|  | Sublay-mesh hernia repair (n=1) | G1: n=1 |
|  | Stomachal fistula repair (n=1) | G1: n=1 |
